# Patient Portals Fail to Collect Structured Information About Who Else is Involved in a Person’s Care

**DOI:** 10.2196/49394

**Published:** 2024-06-27

**Authors:** Liz Salmi, Danielle Peereboom, David A Dorr, Leilani R Graham, Jennifer L Wolff, Catherine M DesRoches

**Affiliations:** 1 Department of General Medicine Beth Israel Deaconess Medical Center Boston, MA United States; 2 Department of Health Policy and Management Johns Hopkins Bloomberg School of Public Health Baltimore, MD United States; 3 Department of Medical Informatics & Clinical Epidemiology Oregon Health & Science University Portland, OR United States; 4 Department of General Medicine Beth Israel Deaconess Medical Center Harvard Medical School Boston, MA United States

**Keywords:** patient portals, shared access, proxy access, portal delegates, caregivers, care partners, health IT, electronic health records

## Abstract

The US health care delivery system does not systematically engage or support family or friend care partners. Meanwhile, the uptake and familiarity of portals to personal health information are increasing among patients. Technology innovations, such as shared access to the portal, use separate identity credentials to differentiate between patients and care partners. Although not well-known, or commonly used, shared access allows patients to identify who they do and do not want to be involved in their care. However, the processes for patients to grant shared access to portals are often limited or so onerous that interested patients and care partners often circumvent the process entirely. As a result, the vast majority of care partners resort to accessing portals using a patient’s identity credentials—a “do-it-yourself” solution in conflict with a health systems’ legal responsibility to protect patient privacy and autonomy. The personal narratives in this viewpoint (shared by permission) elaborate on quantitative studies and provide first-person snapshots of challenges faced by patients and families as they attempt to gain or grant shared access during crucial moments in their lives. As digital modalities increase patient roles in health care interactions, so does the importance of making shared access work for all stakeholders involved—patients, clinicians, and care partners. Electronic health record vendors must recognize that both patients and care partners are important users of their products, and health care organizations must acknowledge and support the critical contributions of care partners as distinct from patients.

## Introduction

As digital tools continue to expand patient roles in health care interactions, technology integrations such as shared access, which uses separate identity credentials for patients and their portal delegates, allow patients to identify who they do and do not want involved in their care. However, clinical teams rarely communicate with patients about the benefits of shared access, and health systems often make registration for these features so onerous that interested patients and their desired delegates circumvent the steps entirely. Health care organizations must acknowledge and support the critical contributions of “portal delegates” as distinct from patients, and electronic health record (EHR) vendors need to make shared access features in portals easier to use for both.

The personal narratives in this viewpoint, shared by DAD, LS, and LRG with permission, elaborate on quantitative studies and provide first-person snapshots of challenges faced by patients and families as they attempt to gain or grant shared access during crucial moments in their lives.

## Shared Access to the Patient Portal

When functioning well, patient portals can place patients in the driver’s seat for their care and improve communication with the health care team. However, portals typically fail to collect structured information about who else is involved in the person’s care, what these individuals do, and their capacity to handle care demands [1]. Caregivers (or “care partners”) play an important role in navigating health system demands, and they commonly provide, oversee, and coordinate medical, nursing, and daily living tasks [1]. Care partners comanage care in myriad ways, such as (1) arranging and scheduling services, (2) joining patients in visits, (3) providing emotional support, and (4) participating in making important care decisions. Care partners are often family members but may also be neighbors, friends, or others having a close relationship with a patient and may or may not be involved in the provision of hands-on assistance with daily activities [2]. Unfortunately, health systems commonly under-engage and lack basic support services for care partners. If services for care partners are available, they are often not covered by insurance. As such, clinicians—facing burnout, staffing shortages, and a growing level of burden with in-basket management—rely on a “shadow workforce” of care partners who are often ill-prepared to assume these roles [3], and reimbursement mechanisms are often insufficient to encourage and support such engagement [4].

According to the Office of the National Coordinator for Health Information Technology (ONC), 86% of US acute care hospitals allow patients to register someone they trust for shared access to a patient portal account with identity credentials (ie, username and password) [5]. Shared access to the portal allows patients to formally identify and involve care partners in their care by designating them as “portal delegates” with their identity credentials. Shared portal access facilitates an information exchange among patients, clinicians, and care partners. It helps clinicians better understand who is involved in health system interactions when it is someone other than a patient, while simultaneously providing care partners with legitimacy and information transparency when engaging in health system activities. However, the uptake of shared access is low, leaving clinicians and other staff guessing about the support available at home or in the community (Textbox 1) [6].

It is important to note that portal delegates are different from health care or medical proxies. A medical proxy is a person who is legally authorized to make decisions on behalf of a person who is physically or mentally unable to make their own medical decisions. Emerging focus group research shows that “shared access” is the preferred term of use for explaining these concepts because it better conveys the intended purpose and functionality of separate login credentials for patients and their portal delegates [7,8].

More structured information about a patient’s informal care team, when placed in the clinical record or integrated through clinical decision support tools [9], could help clinical teams understand who may help the patient at home or in the community (such as social organizations, religious groups, or neighbors) and provide context about who a patient trusts with knowledge of their health and information.

The following personal narratives, shared by permission, elaborate on quantitative studies and provide first-person snapshots of challenges faced by patients and portal delegates as they attempt to gain or grant shared access during crucial moments in their lives.

**Defining shared access.**

- Shared access is sometimes referred to as proxy access.
- Shared access is different from a health care or medical proxy—which is a person legally authorized to make decisions on behalf of a person who is physically or mentally unable to make their own medical decisions.
- Shared access provides portal delegates with legitimacy and information transparency when engaging in health system activities about a patient’s health and their treatments.
- Shared access can help clinicians better understand who is involved in health system interactions when it is someone other than a patient.
- When used as designed, shared access facilitates information exchange between patients, clinicians, and portal delegates.
- Getting shared access to work well for patients, portal delegates, and clinicians is critically important as digital modalities become the mainstream mode of interaction in health care.
- Shared access is especially important for patients who have lower digital health literacy and less experience with health tools, which are increasingly being moved online.

## A Care Partner Perspective (DAD)

My mother and aunt both receive excellent care at major medical centers that use the same EHR vendor. The only difference is that my aunt’s health system realized that not all portal delegates are patients in their system, and they made the experience of being a delegate easy. In contrast, my mother’s health system made a deliberate choice to make shared access the responsibility of individual clinics.

Within a year, my mother and aunt were both diagnosed with complex medical conditions. As a physician and informaticist, I recommend patients and their care partners register for patient portals so they can easily communicate with their medical team, read visit notes, and view lab results. You would think with today’s technology patient portals would be a breeze to navigate, but from a technical perspective, my mother’s and aunt’s portals could not have been more different.

I attempted to talk my mother through the shared access authorization process to register me as her delegate by having her use a desktop computer, but she could not get it to work. I tried to help by accessing her portal through a mobile app. This time it was me who could not get it to work. We eventually called her doctor’s office; they did not know what to do either. In the end, we learned my mother would need to visit a clinic in person to initiate a “nonpatient” request on my behalf. We tried this with the clinic staff but, despite sending reminder emails to me, it never allowed me to fully log in.

Compare this with my aunt who lives quite far away from me. As the doctor in our family, I help remotely whenever I can. A few clicks around her health system’s website revealed my aunt could grant shared access to me as her delegate even though I was not a patient at her hospital. The solution was handled electronically. It was elegant and trouble free. I was surprised and impressed.

If medical professionals are truly committed to advancing shared access, we must be open to learning even more efficient ways of doing things and committed to innovation.

## A Patient Perspective (LS)

Brett became my care partner at a relatively young age after I was diagnosed with a malignant brain tumor at the age of 29 years. He was witness to 2 brain surgeries before we married in the middle of a 2-year stint of chemotherapy.

Ten years later, my tumor recurred. I was scheduled for a third brain surgery, and we had 2 weeks to prepare. I knew the importance of having Brett officially registered as my portal delegate, but the hospital I trust with operating on my brain is 2 hours away and required a wet signature. On top of getting everything else in my life in order in the days leading to brain surgery, I spent hours calling my doctor’s office, sending emails, and sitting on hold in an attempt to grant my husband shared access. I fruitlessly fiddled with my iPhone (Apple Inc) and attempted to send pictures of my signature authorizing my spouse as a portal delegate. According to the hospital, nothing short of a wet signature is acceptable.

Out of necessity and frustration, I circumvented formal channels by manually changing my portal settings to send immediate email and text alerts directly to my husband’s email address. This made it possible for Brett to receive real-time notifications for every test result and progress note.

My over-preparedness and advanced digital planning turned out to be a blessing. The surgery left me with unexpected neurological deficits I am still learning to navigate today. It became hard for me to process numbers and understand dates and times. Making sense of medications and dosing, and tracking appointments became a challenge. Rather than turning off text and email alerts as originally intended, we remained subscribed, which permitted us to not worry about missed messages. I asked Brett if the alerts were information overload, but he believes it is worth it so he can make sure nothing falls through the cracks.

## A Young Adult Perspective (LRG)

In the beginning, having access to my medical record at my fingertips was just annoying.

“I can’t have an outpatient echo today; I’m literally in the ICU,” I told my mother, as I silenced yet another appointment reminder notification.

My unscheduled heart transplant at the age of 24 years nullified the need for routine testing of my native, diseased heart. However, my transition from being cared for by the heart failure clinic to the exclusive property of the transplant team was not communicated between electronic medical record systems.

“I will ask someone to let them know,” my mother soothed. At this stage in my recovery, I did not yet know how to read lab values or translate procedure jargon. I was not yet able to communicate to my usual degree, and telling people, “I no longer have that heart,” was not as straightforward a declaration as I believed it to be.

However, the second I was discharged from the hospital, circumstances forced my hand (rather, all of me) to become a vessel of amateur medical knowledge. Staying alive was a full-time job, and the patient portal became an operating manual. Over time, this access has brought me a great sense of peace, inclusion, and partnership with my care team.

Barely a month after my stay, my dad was admitted to the hospital for a cardiac procedure. Although less severe, my father’s heart developed from the same genetic mutation he passed on to me. We do not, however, share the same desire to be medically literate. After repeated unsuccessful attempts to glean a level of detail from his clinic visit recaps, I insisted on conferencing in, often dominating the conversation with my questions.

“Lei,” he finally said. “Why don’t I just sign something to give you full access to my medical record? You had the disease. You know what needs to be asked.”

Using my patient portal made me a better patient and a stronger advocate. Being made a portal delegate to my dad’s record has made me a better daughter.

## Shared Access Honors Patient Health Information Preferences

As demonstrated in these narratives, adults of all ages commonly rely on and desire care partner involvement in their care, which is facilitated by access to timely and accurate health information [10-12]. Shared access to patient portals can facilitate and improve patient and care partner experiences of care; patient and care partner insight into patient health, activation, and continuity of care; and the ability to engage in health system interactions and the management of tasks [13-16]. However, the ease of shared access registration varies widely even though the functionality exists across health systems [17].

When used as designed, shared access facilitates information exchange between patients, clinicians, and their portal delegates. Shared access helps clinicians better understand with whom they are interacting when it is someone other than the patient. It ensures patient health information preferences are honored while being able to provide care partners with legitimacy for their digital interactions and easy access to their loved one’s health information. Shared access is especially important for patients with lower levels of digital health literacy and less experience with tools that are increasingly being moved online.

As illustrated by DAD and LS, the processes for patients to grant shared access to a portal delegate can be so limited or onerous that interested patients and delegates circumvent the process entirely. Instead, most potential delegates access the portal using the patient’s identity credentials by sharing usernames and passwords—a “do-it-yourself” solution in conflict with a health system’s legal responsibility to protect patient privacy and autonomy [13,18-21]. For example, a study by Pecina et al [20] looked at messages sent to a single health system through an online portal over a calendar year (n=752,551). Of these messages, the study found that 99.3% of messages were sent using the patient’s identity credentials, and 0.7% were sent using a portal delegate’s identity credentials [20]. The study team reviewed 3000 randomly selected portal messages and found that 83.8% (n=2512) were sent by the patient; 7.4% (n=221) were sent from someone other than the patient (indicated by the use of a third-person pronoun, eg, “John needs a refill on his medication”); and in 8.9% (n=266) of messages, the identity of the sender was unclear [20] (Table 1). A recent scoping review of the published literature found that in 7 articles, <3% of adult patient portal accounts were identified as having 1 or more formally registered portal delegates [16].

**Table 1:** Messages sent via the patient portal: from the patient, from someone other than the patient, and unclear of sender (adapted from Pecina et al [20]).

Messages sent via the patient portal	Message sent from a patient, n (%)	Message sent from someone other than the patient, n (%)	Unclear whether the message was sent by a patient or someone else, n (%)
Identity credentials used	747,581 (99.3%)	4970 (0.7%)	—^a^
Text analysis results	2512 (83.8%)	221 (7.4%)	266 (8.9%)

^a^Not available.

Sharing usernames and passwords between patients and care partners is an undesirable practice for several reasons. First, it compromises clinician-patient confidentiality, as clinicians can often identify when the message sender is not the patient, leading to confusion and mistrust. This concern is amplified for older adults who may lack digital proficiency. This practice undermines data integrity and accountability and potentially leads to misinterpretation or misuse of health information. In addition, sharing credentials conflicts with legal and ethical regulations, such as the Health Insurance Portability and Accountability Act (HIPAA), which could jeopardize patient privacy and health care provider obligations [22].

While shared access holds potential as a powerful tool, raising awareness and uptake, and getting shared access to work well for all parties involved, is critically important as digital modalities become the mainstream mode of interaction in health care [23]. In the patient narratives, LRG and LS highlighted the importance of the ability to update portal delegate credentials during crucial moments. Ensuring that all patients and care partners can access these options when they need it will require maintained effort by EHR vendors and health care organizations.

First, the process of requesting and granting shared access must be streamlined, and EHR vendors should work to make the electronic process more intuitive for patients and care partners by incorporating human-centered design principles into the portal interface [24]. As shown through DAD’s story, health care organizations should remove barriers such as requiring that patients request shared access in person and recognize that portal delegates may not be patients in the same health care system or even live in the same geographic area as the patient.

Second, health care organizations need to make organizational commitments to strategies that enhance awareness and adoption of shared access, including education of patient communities and health care teams. Because shared access is so rarely used, awareness is quite low among patients, care partners, and clinical teams. The availability of shared access should be prominently described in print and web-based information about the patient portal. The shared access registration process should be clearly detailed with organizational contact information when people have questions (eg, phone number and email) and include working links to the documentation necessary for the patient authorization and delegate identity credentials. Accreditation organizations, such as the Joint Commission, could further spread these practices by recognizing organizations that prioritize shared access. Initiatives such as the Institute for Healthcare Improvement’s Age-Friendly Health Systems and “dementia-capable” systems could recommend shared access as a standard to support care partners of all ages [25-27]. Furthermore, conversations around assigning portal delegates are best practices that apply to adults of any age.

Third, EHR vendors should offer granular privacy controls to meet the needs of various patient or care partner dyads. Health care organizations currently make “one size fits all” decisions about what portal delegates can access through the portal. Similar to the way most portals allow patients to set preferences about notifications, patients could decide what their portal delegate can see or do with their records. These granular privacy controls could respect patient autonomy and place decision-making power in their hands.

Finally, as illustrated by these stories, prioritizing care partner access to the patient’s electronic health information while supporting patient autonomy will require new educational efforts and technology changes that require culture change at a fundamental level. EHR vendors will need to respect that both patients and portal delegates are important users of their products, and health care organizations will need to acknowledge and support the critical contributions of care partners as distinct from patients. There is a growing recognition that everyone is affected by health and functional setbacks that necessitate care partner involvement. It is well past time to acknowledge this reality by making it easier for care partners to get the information they need and to communicate with the care team.

## Ethical Considerations

According to the Beth Israel Deaconess Medical Center (BIDMC) Committee on Clinical Investigations, viewpoints coauthored with individuals representing patient or family perspectives are not human participants’ research. In addition, none of the individuals sharing personal perspectives in this viewpoint are patients within the institutions named in the author list, and informed consent has been waived. No authors representing patient or care partner perspectives were offered compensation for their views.

## Abbreviations

BIDMC: Beth Israel Deaconess Medical Center
EHR: electronic health record
HIPAA: Health Insurance Portability and Accountability Act
ONC: Office of the National Coordinator for Health Information Technology
